# A study on quantitative structure–activity relationship and molecular docking of metalloproteinase inhibitors based on L-tyrosine scaffold

**DOI:** 10.1186/s40199-015-0111-z

**Published:** 2015-04-29

**Authors:** Maryam Abbasi, Fatemeh Ramezani, Maryam Elyasi, Hojjat Sadeghi-Aliabadi, Massoud Amanlou

**Affiliations:** Department of Medicinal Chemistry, Faculty of Pharmacy, Pharmaceutical Science Research Center, Tehran University of Medical Science, Tehran, Iran; Department of Medicinal Chemistry, Faculty of Pharmacy, Isfahan University of Medical Sciences, 81746-73461 Isfahan, Iran; Medicinal & Natural Product Chemistry Research Center, Shiraz University of Medical Sciences, Shiraz, Iran

**Keywords:** GA-PLS, Metalloproteinase inhibitors, MLR, Molecular docking, QSAR

## Abstract

**Background:**

MMP-2 enzyme is a kind of matrix metalloproteinases that digests the denatured collagens and gelatins. It is highly involved in the process of tumor invasion and has been considered as a promising target for cancer therapy. The structural requirements of an MMP-2 inhibitor are: (1) a functional group that binds the zinc ion, and (2) a functional group which interacts with the enzyme backbone and the side chains which undergo effective interactions with the enzyme subsites.

**Methods:**

In the present study, a QSAR model was generated to screen new inhibitors of MMP-2 based on L–hydroxy tyrosine scaffold. Descriptors generation were done by Hyperchem 8, DRAGON and Gaussian98W programs. SPSS and MATLAB programs have been used for multiple linear regression (MLR) and genetic algorithm partial least squares (GA-PLS) analyses and for theoretical validation. Applicability domain of the model was performed to screen new compounds. The binding site potential of all inhibitors was verified by structure-based docking according to their binding energy and then the best inhibitors were selected.

**Results:**

The best QSAR models in MLR and GA-PLS were reported, with the square correlation coefficient for leave-one-out cross-validation (Q^2^_LOO_) larger than 0.921 and 0.900 respectively. The created MLR and GA-PLS models indicated the importance of molecular size, degree of branching, flexibility, shape, three-dimensional coordination of different atoms in a molecule in inhibitory activities against MMP-2.

The docking study indicated that lipophilic and hydrogen bonding interactions among the inhibitors and the receptor are involved in a ligand-receptor interaction. The oxygen of carbonyl and sulfonyl groups is important for hydrogen bonds of ligand with Leu82 and Ala83. R_2_ and R_3_ substituents play a main role in hydrogen bonding interactions. R_1_ is sited in the hydrophobic pocket. Methylene group can help a ligand to be fitted in the lipophilic pocket, so two methylene groups are better than one. The Phenyl group can create a π-π interaction with Phe86.

**Conclusions:**

The QSAR and docking analyses demonstrated to be helpful tools in the prediction of anti-cancer activities and a guide to the synthesis of new metalloproteinase inhibitors based on L-tyrosine scaffold.

## Introduction

The matrix metalloproteinases (MMPs) function predominantly as enzymes that degrade structural components of the extracellular matrix (ECM) [1-4]. MMPs play a substantial role in tumor progression and invasion of inflammatory cells. Among MMPs, MMP-2 easily digests the denatured collagens and gelatins [5,6]. It is highly involved in the process of tumor invasion and has been considered as a promising target for cancer therapy [3,7,8]. MMP-2 has a catalytic center) zinc (II) ion (and two hydrophobic domains (S1´ pocket and S1 pocket). S1´ pocket, the key domain of MMP-2, is deeper and narrower than other MMP subtypes and S1 pocket is a solvent exposure domain [3,9,10].

The structural requirements of an MMP-2 inhibitor are: (1) a functional group that binds the zinc ion (zinc-binding group; ZBG), capable of chelating the active site zinc ion; (2) a functional group which interacts with the backbone of enzyme; (3) side chains that undergo effective interactions with the enzyme subsites, such as S1´ pocket and S1 pocket [3,11,12].

Cheng et al. studied the L–hydroxy proline scaffold-based MMP-2 inhibitors in 2008 [13], and, in order to identify more potent MMP-2 inhibitors, replaced L-hydroxy proline with the L-tyrosine scaffold to form a new integrated structural pattern. They reported that the alteration in substitution pattern at R_1_, R_2_ and R_3_ positions alter MMP-2 inhibitory activity [1].

In 2012, 30 L–hydroxy tyrosine scaffold-based MMP-2 inhibitors were identified. It seems that finding a relationship between the structure of these compounds and their inhibitory activities in order to design structures with better activities and to predict their activity would be essential.

Quantitative structure activity relationships (QSARs), one of the most extensively used methods in chemometrics, and molecular docking are two of the helpful methods for drug design and prediction of drug activity [14-16]. QSAR models are mathematical equations which generate a communication between chemical structures and their biological activities, while molecular docking is done to specify the structural features that are important for interaction with a receptor.

In this report, we have performed a QSAR study and a molecular docking examination on 30 compounds of L-tyrosine derivatives which had been synthesized and evaluated as metalloproteinase (MMP-2) inhibitors [1].

## Materials and methods

### QSAR

All calculations were implemented using an Intel Core i5 2.4 GHz processor, with the windows 7 operating system. Geometry optimization was done by Hyperchem 8.0 software. Descriptor generation was performed by Hyperchem 8.0, DRAGON package and Gaussian 98 W programs. SPSS software (version 11.5) and MATLAB software (version 7.12.0) were used for model creation and validation methods.

### Activity data and descriptors generation

In this study, the biological data employed is MMP-2 inhibitory activity of 30 compounds. The synthesis and determination of biological activity of these inhibitors have been reported by Cheng et al. [1]. The structure of these compounds and their biological activity are shown in Table 1. The two-dimensional structures of molecules were drawn using Hyperchem 8.0 software. At the beginning, pre-optimization was conducted using the MM+ molecular mechanic force field and then a more accurate optimization was performed with the semi-empirical PM3 method. The optimization was performed using the Polak–Ribiere algorithm until the root mean square gradient reached 0.01 kcal/ (Å mol). Hyperchem 8.0 program was also used to calculate chemical descriptors including: surface area, molecular volume, hydration energy, octanol/water partition coefficient (logP), molar refractivity, molar polarisability and molar mass.

**Table 1:** **Chemical structures of L-tyrosine derivatives and their experimental and predicted activity by MLR and GA-PLS models**


**Comp.**	**R**_**1**_	**R**_**2**_	**R**_**3**_	**Experimental pIC**_**50**_	**Predicted pIC**_**50**_	
					**MLR**	**GA-PLS**
4a	C_6_H_5_CH_2_	C_6_H_5_CO	OCH_3_	4.17	4.01	3.90
4b	C_6_H_5_CH_2_	p-CH_3_C_6_H_4_CO	OCH_3_	4.08	3.82	3.92
4c	C_6_H_5_CH_2_	CH_3_CO	OCH_3_	4.56	4.42	4.61
4d	C_6_H_5_CH_2_	CH_3_SO_2_	OCH_3_	4.75	4.74	4.74
4e	C_6_H_5_CH_2_	p-CH_3_C_6_H_4_SO_2_	OCH_3_	4.97	4.75	4.86
4f	C_6_H_5_CH_2_CH_2_	C_6_H_5_CO	OCH_3_	4.13	4.16	3.79
4 g	C_6_H_5_CH_2_CH_2_	p-CH_3_C_6_H_4_CO	OCH_3_	4.07	3.94	4.42
4 h	C_6_H_5_CH_2_CH_2_	CH_3_CO	OCH_3_	4.90	4.98	4.97
4i	C_6_H_5_CH_2_CH_2_	CH_3_SO_2_	OCH_3_	5.62	5.82	5.98
4j	C_6_H_5_CH_2_CH_2_	p-CH_3_C_6_H_4_SO_2_	OCH_3_	5.20	5.24	5.44
5a	C_6_H_5_CH_2_	C_6_H_5_CO	OH	5.01	4.39	4.78
5b	C_6_H_5_CH_2_	p-CH_3_C_6_H_4_CO	OH	5.09	4.77	4.3
5c	C_6_H_5_CH_2_	CH_3_CO	OH	5.52	5.68	5.66
5d	C_6_H_5_CH_2_	CH_3_SO_2_	OH	5.85	6.03	6.06
5e	C_6_H_5_CH_2_	p-CH_3_C_6_H_4_SO_2_	OH	5.60	5.99	5.91
5f	C_6_H_5_CH_2_CH_2_	C_6_H_5_CO	OH	5.12	5.57	5.33
5 g	C_6_H_5_CH_2_CH_2_	p-CH_3_C_6_H_4_CO	OH	5.35	5.90	5.82
5 h	C_6_H_5_CH_2_CH_2_	CH_3_CO	OH	6.12	6.67	6.08
5i	C_6_H_5_CH_2_CH_2_	CH_3_SO_2_	OH	6.92	6.69	6.48
5j	C_6_H_5_CH_2_CH_2_	p-CH_3_C_6_H_4_SO_2_	OH	6.57	6.49	6.52
6a	C_6_H_5_CH_2_	C_6_H_5_CO	NHOH	5.77	6.04	5.84
6b	C_6_H_5_CH_2_	p-CH_3_C_6_H_4_CO	NHOH	6.34	6.42	6.82
6c	C_6_H_5_CH_2_	CH_3_CO	NHOH	7.43	7.32	6.84
6d	C_6_H_5_CH_2_	CH_3_SO_2_	NHOH	7.17	7.44	7.39
6e	C_6_H_5_CH_2_	p-CH_3_C_6_H_4_SO_2_	NHOH	7.60	7.19	7.14
6f	C_6_H_5_CH_2_CH_2_	C_6_H_5_CO	NHOH	6.05	6.05	6.86
6 g	C_6_H_5_CH_2_CH_2_	p-CH_3_C_6_H_4_CO	NHOH	5.41	5.57	5.95
6 h	C_6_H_5_CH_2_CH_2_	CH_3_CO	NHOH	7.89	7.55	7.53
6i	C_6_H_5_CH_2_CH_2_	CH_3_SO_2_	NHOH	7.54	7.91	7.94
6j	C_6_H_5_CH_2_CH_2_	p-CH_3_C_6_H_4_SO_2_	NHOH	7.77	7.49	7.73

The output files of Hyperchem 8.0 software (.hin files) were transferred to DRAGON package to calculate four classes of descriptors (0D, 1D, 2D and 3D) including 28 constitutional descriptors, 10 functional groups, 18 atom-centered fragments, 216 topological descriptors, 15 molecular walk counts, 64 BCUT descriptors, 24 Galvestopol.charge indices, 96 2D autocorrelations, 14 charge descriptors, 41 Randic molecular profiles, 27 geometrical descriptors, 150 radial distribution function descriptors (RDF), 160 3D-MoRSE descriptors, 99 WHIM descriptors and 196 GETAWAY descriptors.

The z-matrix files of compounds were also provided by Hyperchem 8.0 program (.zmt files) and then were transferred as input files to the Gaussian98W program [17]. Semi-empirical molecular orbital calculation by PM3 method was performed using Gaussian98W program. Different quantum chemical descriptors were obtained by this method including highest occupied molecular orbital energy (E_HOMO_), lowest unoccupied molecular orbital energy (E_LUMO_), and molecular dipole moment. Local charge was obtained by PM3 method in Gaussian98W. Hardness (η = 0.5 (E_HOMO_ + E_LUMO_)), softness (S = 1/η), electronegativity (χ = 0.5 (E_HOMO_ - E_LUMO_)) and electrophilicity (ω = χ^2^/2η) were calculated according to the method proposed by Thanikaivelan et al. [18].

### Data processing and models building

All calculated descriptors were collected in a data matrix, D, the number on rows was representative of molecule numbers and the numbers on columns accounted for descriptors (30 × 1168). At the beginning, the columns which had constant and near constant values were removed from the original data matrix. Since collinear variables disrupt the models in MLR analysis, collinear descriptors needed to be detected and removed. The correlation of descriptors with one another and with activity data was then investigated. In the pairs with collinearity higher than 0.9, one which had the highest correlation versus the activity was retained and the rest were omitted. The number of total descriptors for each molecule reached 291.

The data set (30 compounds) was split into a calibration set and validation set. Validation subset was made of 20% of the total data (here, 6 biological activity data). An MLR analysis was performed by the stepwise regression SPSS (version 12.0) software and the model was built.

Since the data splitting has a considerable influence on the final selected model, a combined data splitting-feature selection (CDFS) strategy was employed [19]. In the CDFS methodology, several subdivisions of calibration and validation set were made (10 times). In each case, the best model was chosen with a correlation coefficient higher than 0.95. The created models were validated by leave-one out (LOO) cross-validation method and Y-randomization test to investigate their predictability.

### Molecular docking

Molecular docking has become an increasingly main tool for drug discovery. Docking is a method which predicts the preferred orientation of one ligand when bound in an active site to form a stable complex. In this study, molecular docking of L-tyrosine derivatives with MMP-2 was studied by AutoDock 4.2 program [20] to find their binding site and the best direction based on the binding energy. Pdb file of MMP-2 was obtained from www.pdb.org (PDB: 3AYU) [21]. All water molecules were deleted, polar hydrogens were added and the Kollman charge [22] was computed. A 120 x 120 x 120 Å size was chosen for the grid box, which covers the whole protein. In all of the ligands, the same as protein, polar hydrogens were added and the Gasteiger charge was computed [23]. After preparation of ligand and protein files, the map files were created. Docking process with 50 runs and maximum number of evaluations 2500000 were performed. The final .dlg files were analyzed and the interaction between ligands and the active site of protein were studied. The ligand-protein interactions were analyzed and visualized by Discovery studio Ver. 3.

## Result and discussion

### Multiple linear regression analysis (MLR)

This study made use of an MLR analysis, as a simple regression method. The stepwise regression (using SPSS software) was also utilized to choose the most relevant set of descriptors for each type of the split data. The model coefficients were calculated using calibration data and then were used to predict the biological activity of validation samples. Several models were constructed by running a typical stepwise regression which is ranked based on calibration correlation coefficient (R^2^c). A model with calibration correlation coefficient higher than 0.9 was selected. The created model needed to be validated. The theoretical validation is generally classified into two groups: internal and external validation. Two of the internal validation methods include Leave-one out cross validation (LOO) and Y-randomization [24]. The offered model was evaluated for both over-fitting and avoidance of chance correlations by the leave-one-out cross-validation (LOO) method and Y-randomization test respectively.

MLR method was repeated several times by different splitting data. In each case, one model was proposed. Leave-one-out cross-validation was performed and lastly, the best ten models were obtained with R^2^ and Q^2^_LOO_ higher than 0.9 in as reported in Table 2. The statistical qualities of models were acceptable and all of them had Q^2^_LOO_ larger than 0.92; hence, the predicted models can make over 92% of variances in the inhibitory activity. In addition, results were acceptable in the prediction sets. The values of prediction correlation coefficient (R^2^_p_) are listed in Table 2 for the ten final models. All of the squared correlation coefficients were higher than 0.90, so the resultant linear models can predict 90% of variances in the inhibitory activity in the ten prediction sets.

**Table 2:** **The best ten models were selected for future analysis in MLR**

**NO.**	**Descriptor used**	**R** ^**2**^_**c**_^**a**^	**S.E** ^**b**^	**R** ^**2**^_**p**_^**c**^	**Q** ^**2**^_**LOO**_^**d**^	**RMS**_**CV**_^**e**^
1	IC1, RDF135e, Mor24m, RDF035u, E3u, RDF120m, Mor15e	0.985	0.177	0.988	0.956	0.250
2	IC1, Mor24m, Mor15e, R7e0, G3p	0.989	0.154	0.90	0.967	0.218
3	IC1, Mor24m, Mor15e, dipx, GATS8p, RDF065m	0.986	0.170	0.952	0.962	0.230
4	IC1, SP20, RDF115m, RDF115e, Mor28e	0.988	0.148	0.972	0.972	0.199
5	IC1, Mor24m, Mor15e, Mor09e, G2u, Mor27u	0.976	0.228	0.951	0.940	0.300
6	IC1, Mor24m, Mor15e, Mor09e, G2u, Mor27u	0.969	0.243	0.962	0.932	0.309
7	IC1, Mor28e, HATS3p, RDF120m, RDF115m	0.987	0.152	0.974	0.969	0.203
8	IC1, Mor28e, HATS3p, RDF115m, RDF120m, G3m	0.978	0.194	0.972	0.952	0.245
9	IC1, Mor24m, RDF135e, RDF035v, RDF135m, Mor26m	0.977	0.223	0.979	0.918	0.354
10	IC1, Mor26m, G3p, GATS8p, HATS7e	0.976	0.214	0.953	0.942	0.284

^a^R^2^_c_ = Correlation Coefficient of calibration set.

^b^S.E = Standard error of regression.

^c^R^2^_p_ = Correlation Coefficient of prediction set.

^d^Q^2^_LOO_ = Leave-one-out cross-validation correlation coefficient.

^e^RMSE_CV_ = Root mean square error of cross validation.

The total number of descriptors, which existed in all the ten models, was 22. These descriptors are briefly described in Table 3. Among these, 3 descriptors were common. Some of the descriptors such as E3u, HATS7e, RDF065m, SP20, RDF115e, G3m and dipx were observed only in one model. The repeated descriptors were IC1, Mor24m and Mor15e. IC1 descriptor existed in all the ten models. IC1 is topological descriptor and Mor24m and Mor15e are 3D-MoRSE ones.

**Table 3:** **Brief description of the descriptors in ten models**

**NO.**	**Name**	**Description**
1	IC1	Information content index (neighborhood symmetry of 1-order)
2	RDF135e	Radial Distribution Function −3.5/ weighted by atomic Sanderson electronegativities
3	Mor24m	3D-MoRSE – signal 24/ weighted by atomic masses
4	RDF035u	Radial Distribution Function −3.5/ unweighted
5	E3u	3rd component accessibility directional WHIM index/ unweighted
6	RDF120m	Radial Distribution Function −12.0/ weighted by atomic masses
7	Mor15e	3D-MoRSE – signal 15/ weighted by atomic Sanderson electronegativities
8	G3p	3st component symmetry directional WHIM index/weighted by atomic polarizabilities
9	R7e0	R maximal autocorrelation of lag 7/weighted by atomic Sanderson electronegativities
10	dipx	Molecular dipole moment at X-direction
11	GATS8p	Geary autocorrelation -lag 8/ weighted by atomic polarizabilities
12	RDF065m	Radial Distribution Function −6.5/ weighted by atomic masses
13	SP20	Shape profile no. 20
14	Mor28e	3D-MoRSE – signal 28/ weighted by atomic Sanderson electronegativities
15	Mor09e	3D-MoRSE – signal 09/ weighted by atomic Sanderson electronegativities
16	G2u	2st component symmetry directional WHIM index/ unweighted
17	Mor27u	3D-MoRSE – signal 27/unweighted
18	RDF115m	Radial Distribution Function −11.5/ weighted by atomic masses
19	RDF115e	Radial Distribution Function −11.5 / weighted by atomic Sanderson electronegativities
20	HATS3p	Leverage- weighted autocorrelation of lag 3/ weighted by atomic polarizabilities
21	G3m	3st component symmetry directional WHIM index/ weighted by atomic masses
22	Mor26m	3D-MoRSE – signal 26/ weighted by atomic masses

The repetition of IC1 in all the ten models indicated that this descriptor has a main effect on L-tyrosine scaffold-based MMP-2 inhibitors. 3D-MoRSE descriptors also have significant effects. The quantum descriptors such as (dipx) that were seen only in one model have lower effects on MMP-2 inhibitors. The observation of the models, as listed in Table 2, revealed that there is a high degree of resemblance between the subset of descriptors (without considering the difference between the chosen descriptors in different models).

To create a general model, all of the 22 descriptors were used and an MLR analysis was applied with the stepwise variable selections. Finally, one model with balance between the highest R^2^, Q^2^_LOO_ and the lowest number of descriptors was opted for further analysis, as reported in MLR Equation 1:

$$ \mathrm{pI}{\mathrm{C}}_{50} = \hbox{-} 13.428\ \left( \pm 1.720\right) + 5.965\ \left( \pm 0.488\right)\ \mathrm{I}\mathrm{C}1 + 1.464\ \left( \pm 0.618\right)\ \mathrm{Mor}24\mathrm{m}\ \hbox{-}\ 0.187\ \left(\pm 0.044\right) $$

1 $$ \mathrm{R}\mathrm{D}\mathrm{F}115\mathrm{m} + 0.307\ \left(\pm 0.067\right)\ \mathrm{S}\mathrm{P}20 + 1.284\ \left(\pm 0.514\right)\ \mathrm{Mor}15\mathrm{e} $$

$$ \mathrm{N} = 24,\ {{\mathrm{R}}^2}_{\mathrm{c}} = 0.958,\ \mathrm{S}.\mathrm{E} = 0.285,\ {{\mathrm{Q}}^2}_{\mathrm{LOO}} = 0.921,\ \mathrm{R}\mathrm{M}{\mathrm{S}}_{\mathrm{CV}} = 0.346 $$

In this equation, N represents the number of molecules in the calibration set. R^2^_c_ and Q^2^_LOO_ are respectively the squared correlation coefficients for calibration and cross-validation. In addition, S.E is the standard error of calibration and RMS_CV_ is the root mean square error of cross-validation. This equation has three common descriptors (IC1, Mor24m and Mor15e), with high calibration statistics and prediction ability.

Topological descriptors, such as IC1, explain the size, degree of branching, flexibility and overall shape. 3D-MoRSE descriptors explain three-dimensional coordination of the different atoms in a molecule. IC1, Mor24m and Mor15e have positive signs which indicate that pIC_50_ is directly related to these descriptors. The radial distribution function (RDF) descriptors are based on the distance distribution in the molecule. RDF115m has a negative sign which indicates that pIC_50_ is inversely related to this descriptor.

For the MMP-2 inhibition activity, a higher value of molecular size, degree of branching, flexibility, shape, three-dimensional coordination of the different atoms in a molecule (IC1) and a lower value of radial distribution function, RDF115m, are beneficial to the activity.

**Figure 1:**
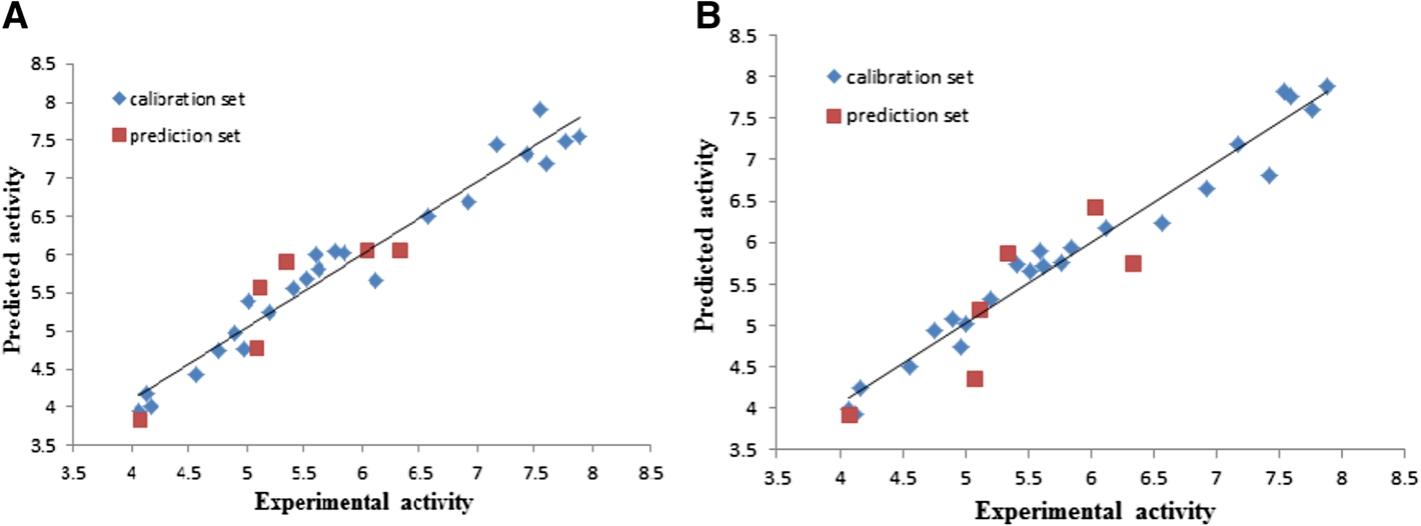
Plot of predicted pIC_50_ versus the experimental values for MLR model **(A)**, and GA-PLS model **(B)**.

The general model has a Q^2^_LOO_ equal to 0.921; hence, the predicted model can make over 92% of variances in the inhibitory activity. The predicted values of pIC_50_ were obtained for all the molecules by MLR equation which was listed in Table 1 and were plotted against the experimental values (Figure 1A).

The Y-randomization test was performed to evaluate the robustness of the general model. In the Y-randomization test, the activity data were randomly permuted in the original model and 10 models were generated. All of the models were expected to have lower R^2^ and Q^2^_LOO_ values than the original MLR model. If the reverse occurs, a suitable MLR model cannot be generated. The lower R^2^ and Q^2^_LOO_ values are shown in Table 4.

**Table 4:** **R** ^**2**^ **and Q** ^**2**^_**LOO**_ **values after ten Y-randomization tests**

**Iteration**	**MLR**		**GA-PLS**	
	**R** ^**2**^	**Q** ^**2**^_**LOO**_	**R** ^**2**^	**Q** ^**2**^_**LOO**_
1	0.204	0.001	0.319	0.102
2	0.048	0.265	0.048	0.244
3	0.314	0.068	0.206	0.016
4	0.075	0.260	0.040	0.235
5	0.118	0.022	0.097	0.028
6	0.106	0.069	0.132	0.020
7	0.256	0.010	0.394	0.194
8	0.096	0.099	0.038	0.296
9	0.068	0.175	0.062	0.175
10	0.191	0.007	0.177	0.002

### Genetic algorithm partial least squares (GA-PLS)

Since the variable selection method, that is the stepwise regression, may not be a suitable selection, variable selection methods such as the genetic algorithm, which demonstrate much better outcomes in comparison with the stepwise regression, are more favorable [25,26].

In genetic algorithm, if its corresponding descriptor is contained, a gene receives a value of 1 in the subset; otherwise, it takes a value of zero. The number of genes at each chromosome is equivalent to the number of descriptors. The population size varied between 80 and 125 for different GA runs. The number of genes with the values of 1 was relatively lower than the number of genes with the values of 0. The genes with the values of 1 were maintained. The chromosomes with the smallest numbers of chosen descriptors (total number of descriptors for each molecule reached 105) and the highest fitness were selected as the intended model. The predicted model was tested by leave-n-out cross-validation [27]. A leave-one-out cross-validation was triggered and the value of Q^2^_LOO_ was obtained 0.850.

In GA-PLS, the resulted model with higher cross-validation statistics is reported in Equation 2 and the predicted values of pIC_50_ are shown in Table 1 and plotted against the experimental values in Figure 1B.

$$ \mathrm{pI}{\mathrm{C}}_{50} = \hbox{-} 12.589\ \left( \pm 1.208\right) + 6.363\ \left( \pm 0.394\right)\ \mathrm{I}\mathrm{C}1 + 2.119\ \left( \pm 0.485\right)\ \mathrm{Mor}24\mathrm{m}\ \hbox{-}\ 0.665\ \left( \pm 0.189\right) $$

2 $$ \mathrm{Mor}15\mathrm{e}\ \hbox{-}\ 0.784\ \left( \pm 0.370\right)\ \mathrm{Mor}32\mathrm{e} $$

$$ \mathrm{N} = 24\kern1.5em {{\mathrm{R}}^2}_{\mathrm{c}} = 0.932\kern1em \mathrm{S}.\mathrm{E} = 0.328\kern1em {{\mathrm{Q}}^2}_{\mathrm{LOO}} = 0.900\kern3.25em \mathrm{RMSCV} = 0.391 $$

This equation has three MLR descriptors (IC1, Mor24m and Mor15e) with high calibration statistics and prediction ability. IC1 and Mor24m have a display positive effect and Mor15e and Mor32e have a display negative effect on inhibitory activity in GA-PLS. Unlike MLR, The radial distribution function (RDF) descriptors have no effect on inhibitory activity in GA-PLS.

As observed from Equation 2, the variables used in this equation can explain 93.2% of the variances in the inhibitory activity of the MMP-2 inhibitors used in this study.

The Y-randomization test was performed to evaluate the robustness of the created model in GA-PLS and 10 models were generated. All of the models were expected to have lower R^2^ and Q^2^_LOO_ values than the original GA-PLS model, as shown in Table 4.

### Applicability domain of the model

**Figure 2:**
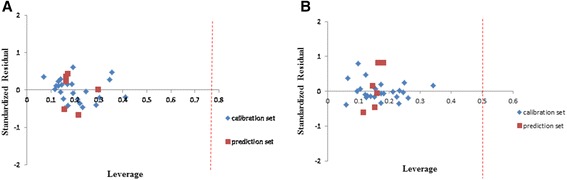
William’s plot of generated MLR model **(A)**, and GA-PLS model **(B)**.

A QSAR model is exploited to screen new compounds when its domain of application has been defined [28]. The prediction may be assumed reliable for only those compounds which fall into this domain [29]. Standardized residuals of the activity were computed and were plotted versus leverage values (h). The value of leverage was calculated for every compound. Values are always between 0 and 1. A value of 0 is indicative of perfect prediction and usually is not accessible, and a value of 1 indicates very poor prediction. The lower the value, the higher confidence in the prediction. Warning leverage (h*) is another standard for explanation of the results and is, generally, fixed at 3 (k + 1)/ n, where k is the number of model parameters and n is the number of calibration set [29]. Calculated leverage for calibration set is useful for determining the compounds which affect the model and, in terms of validation set, useful for assigning the applicability domain of the model. The William’s plot for the developed models in MLR and GA-PLS are shown in Figure 2.

Response outliers are compounds that have standard residual points higher than ± 2.0 standard deviation units and a leverage value higher than the warning leverage, which is 0.75 and 0.5 for MLR and GA-PLS respectively. As can be seen in Figure 2, all studied molecules in calibration and validation set lie with high degree of confidence in application domain of the developed models in both methods.

### Molecular docking studies

**Figure 3:**
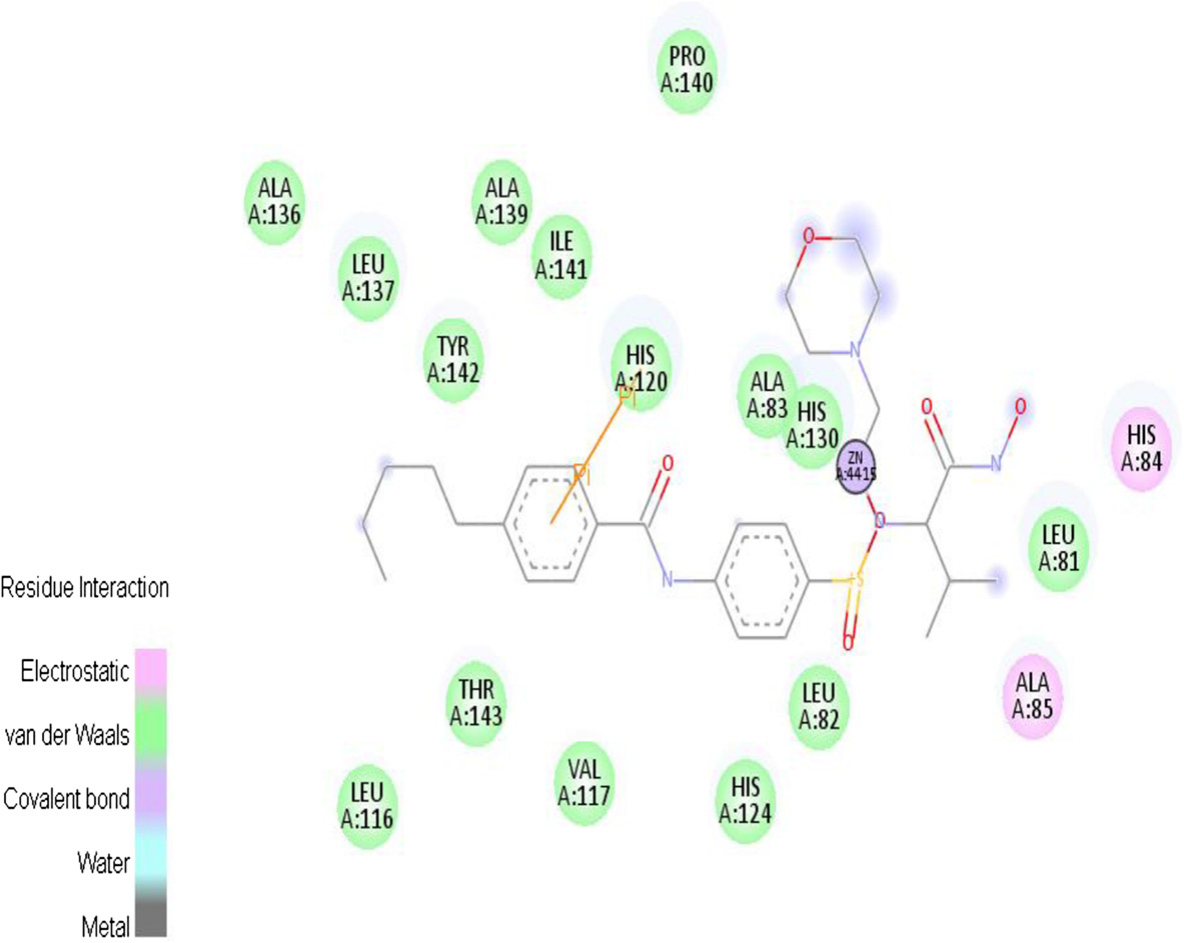
Interactions between ligand and MMP-2.

To explore the ligand binding modes, and to find amino acids, which are essential in ligand binding to MMP-2, molecular docking was performed on a ligand-binding pocket. The way the compound was bound with the lowest free energy was studied. Interactions between MMP-2 and ligand N-4-[(1-hydroxycarbamoyl-2-methyl-propyl)-(2-morpholin-4-yl-ethyl)-sulfamoyl]-4- pentyl-benzamide (SC-74020) were obtained by Feng et al. and were reported. According to their results, the catalytic zinc is chelated by His120, His124, His130 and ligand, and the structural zinc is coordinated by His70, Asp72, His85 and His98. In addition, hydrogen bond was bound by Leu82 and Ala83 to a sulfoneoxygen atom of the inhibitor [30]. Initially, to assure binding mode of ligand and protein, ligand docking with MMP-2 protein has been validated by Feng. All of the interactions between the ligand and catalytic zinc with the protein from our results are shown in Figure 3. Root mean square deviation (RSMD) was lower than 1. Both of the prior cases proposed high reliability of docking program. Therefore, the AutoDock 4.2 could be used to find the binding mode of other inhibitors of MMP-2.

**Figure 4:**
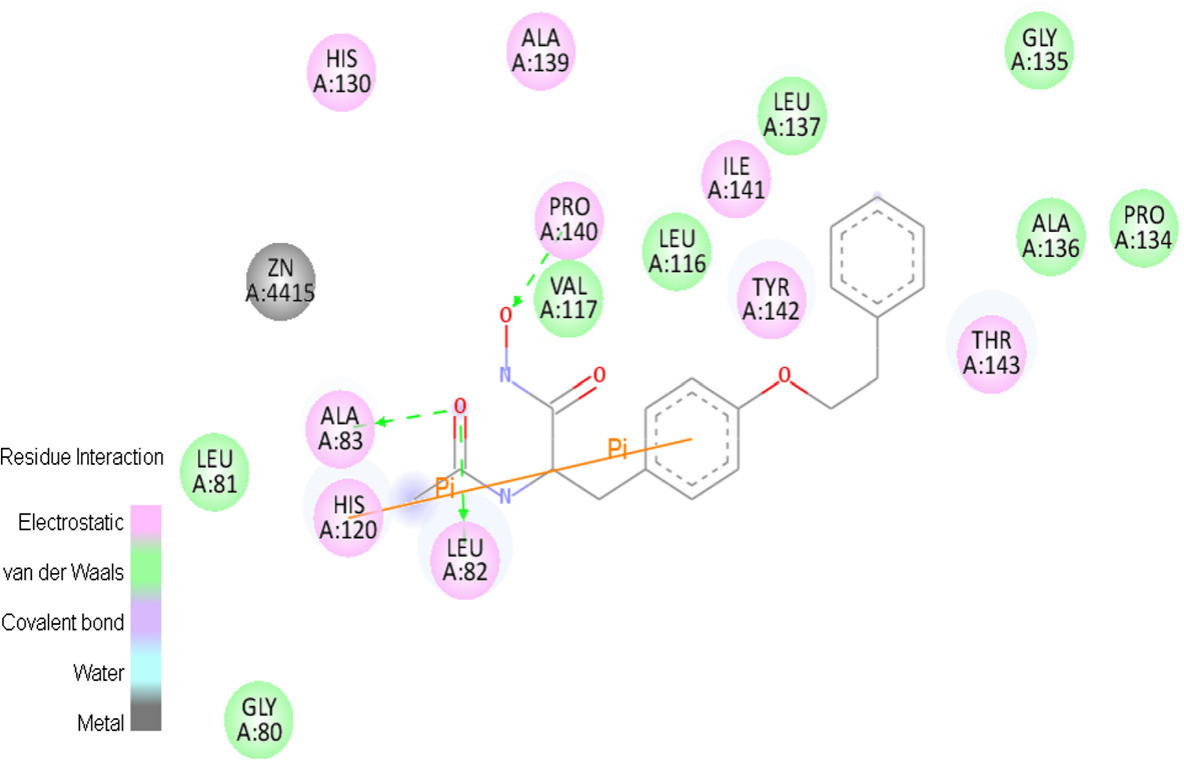
The best orientation of 6 h ligand.

All of the 30 compounds were docked into the binding site of protein by AutoDock 4.2 and were studied. The binding energy of all the compounds is reported in Table 5. The obtained energies were compared with the experimental IC_50_, and the 6a-6j compounds have the lowest binding energy. This means that based on the binding energy of the active site, these compounds, especially compound (6 h), are the best L-tyrosine scaffold based inhibitors. The binding orientation of compound (6 h) and hydrogen bond of the ligand with Leu82 and Ala83 are depicted in Figure 4. This compound was fitted into the active site of MMP-2. In all of the compounds, the oxygen of carbonyl and sulfonyl groups are important for hydrogen bond of the ligand with Leu82 and Ala83. R_2_ and R_3_ substituents have the main role for hydrogen bonding interactions. In 4a-4j compounds, IC_50_ is higher than 5a-5j and 6a-6j compounds. When R_3_ is OH and NHOH groups, hydrogen bond can be created better than when R_3_ is –OCH_3_. Moreover, when R_2_ is sulfonyl, because of two oxygen groups, a stronger hydrogen bond can be created than that of carbonyl.

**Table 5:** **The obtained binding energy by AutoDock**

**Compound**	**Binding energy (kcal/mol)**	**Compound**	**Binding energy (kcal/mol)**
4a	−5.90	5f	−6.13
4b	−5.57	5 g	−6.34
4c	−5.96	5 h	−5.93
4d	−5.39	5i	−5.92
4e	−6.88	5j	−6.79
4f	−5.88	6a	−7.08
4 g	−5.67	6b	−7.07
4 h	−6.23	6c	−7.01
4i	−6.21	6d	−7.27
4j	−5.98	6e	−7.29
5a	−6.68	6f	−7.16
5b	−6.76	6 g	−6.92
5c	−6.85	6 h	−7.35
5d	−6.47	6i	−7.30
5e	−6.67	6j	−7.29

R_1_ is sited in hydrophobic pocket. Methylene group can help the ligand to be fitted in lipophilic pocket, so two Methylene groups are better than one. Phenyl group can create a π- π interaction with Phe86. Therefore, phenethyl (C_6_H_5_CH_2_CH_2_) is better than benzyl (C_6_H_5_CH_2_).

To design L-tyrosine based inhibitors and prediction of their activity against MMP-2 based on AutoDock 4.2 and QSAR studies, addition to a higher value of molecular size, degree of branching, flexibility, shape and three-dimensional coordination of the different atoms in a molecule, hydrophobicity and hydrophilicity of R_1_, R_2_ and R_3_ are highly important. When R_1_ is more hydrophobic, and R_2_, is more hydrophilic, there is a stronger inhibition against MMP-2.

## Conclusion

In this study, quantitative relationships between molecular structure and inhibitory effect of L-tyrosine scaffold based MMP-2 inhibitors were investigated by MLR and GA-PLS. In MLR, A combined data splitting-feature selection method (CDFS) was offered to develop a quantitative structure–activity relationship model. It was found that topological parameter (IC1) has a main effect on the inhibitory activity of the compounds, among the different QSAR models. By this simple procedure, a multilinear 5-parametric model was created out of 22 descriptors. This method yielded acceptable results for the prediction set which was measured by cross-validation and Y-randomization. The findings indicate that the linear model produced by CDFS methodology could reproduce more than 92% of variances in the inhibitory activity. In addition to MLR, genetic algorithms, which demonstrated much better outcomes in comparison with stepwise regression, were used. By GA-PLS, the model with higher cross-validation statistics was created and the results indicated that IC1, Mor24m, Mor15e and Mor32e are main descriptors. It can be concluded from the two methods that higher values of molecular size, degree of branching, flexibility, shape and three-dimensional coordination of the different atoms in a molecule are particularly important

The docking study revealed that two hydrogen bonds between all of the inhibitors and the active site of MMP-2 (Leu82 and Ala83) are formed, as well as a π-π interaction between phenyl group and Phe86.

The information above could be used to design new inhibitors, and to show higher inhibitory activities and chemical synthesis of new inhibitors.

## Abbreviations

MMP: Matrix metalloproteinase
QSAR: Quantitative structure activity relationship
MLR: Multiple linear regression
ECM: Extracellular matrix
CDFS: Combined data splitting-feature selection
Q^2^_LOO_: Square correlation coefficient for leave-one-out cross-validation
R^2^_c_: Calibration correlation coefficient
R^2^_p_: Prediction correlation coefficient
RSMD: Root-mean-square deviation
S.E: standard error of calibration
RMS_CV_: root mean square error of cross-validation
RDF: radial distribution function descriptors
GA-PLS: Genetic algorithm partial least squares
